# Now and then: Hand choice is influenced by recent action history

**DOI:** 10.3758/s13423-018-1510-1

**Published:** 2018-07-23

**Authors:** Kenneth F. Valyear, Aoife M. Fitzpatrick, Neil M. Dundon

**Affiliations:** 1 School of Psychology, Bangor University, 355 Brigantia Building, Bangor, Gwynedd, Wales LL57 2AS UK; 2 Brain Imaging Center, Department of Psychological and Brain Sciences, University of California, Santa Barbara, Santa Barbara, CA USA; 3 Department of Child and Adolescent Psychiatry, Psychotherapy and Psychosomatics, University of Freiburg, Freiburg, Germany

**Keywords:** Hand choice, Motor history, Priming, Action selection, Action planning, Hysteresis, Motor programming, Sensorimotor control

## Abstract

Action choices are influenced by recent past and predicted future action states. Here, we demonstrate that recent hand-choice history affects both current hand choices and response times to initiate actions. Participants reach to contact visible targets using one hand. Hand choice is biased in favour of which hand was used recently, in particular, when the biomechanical costs of responding with either hand are similar, and repeated choices lead to reduced response times. These effects are also found to positively correlate. Participants who show strong effects of recent history on hand choice also tend to show strong effects of recent history on response times. The data are consistent with a computational efficiency interpretation whereby repeated action choices confer computational gains in the efficiency of underpinning processes. We discuss our results within the framework of this model, and with respect to balancing predicted gains and losses, and speculate about the possible underlying mechanisms in neural terms.

**Electronic supplementary material:**

The online version of this article (10.3758/s13423-018-1510-1) contains supplementary material, which is available to authorized users.

Hand choice is influenced by a range of factors, including predicted differences in biomechanical and energetic consequences (Bryden & Huszczynski, 2011; Habagishi, Kasuga, Otaka, Liu, & Ushiba, 2014; Schweighofer, Xiao, Kim, Yoshioka, Gordon, & Osu, 2015), performance metrics (Coelho, Przybyla,Yadav, & Sainburg, 2013; Kim, Buchanan, & Gabbard, 2011), and success likelihood (Stoloff, Taylor, Xu, Ridderikhoff, & Ivry, 2011). Choices tend to reflect those that provide effective performance with minimal costs. For example, reaching to different areas of space is associated with different energetic costs related to the inertial properties of the arm (Gordon, Ghilardi, Cooper, & Ghez, 1994). Under conditions of free choice, both hand (Schweighofer et al., 2015) and arm-movement (Cos, Belanger, & Cisek, 2011; Dounskaia, Goble, & Wang, 2011; Sabes & Jordan, 1997) choices respect these constraints. These data are consistent with leading accounts of action selection that stress the importance of balancing predicted gains and losses (Elsinger & Rosenbaum, 2003; Shadmehr, Huang, & Ahmed, 2016).

Hand choice is also influenced by recently performed actions. Schweighofer et al. (2015) identify hand-use history as a significant predictor of hand choice, alongside estimated limb-specific energetic costs and success likelihood. Other studies also demonstrate effects of recent action history on hand choice (Rostoft, Sigmundsson, Whiting, & Ingvaldsen., 2002; Weiss & Wark, 2009). Hand choice is biased in favour of the hand that was used recently. Consistent with these data, recent action history also affects grasp choices (Cohen & Rosenbaum, 2004, 2011; Dixon, McAnsh, & Read, 2012; Kelso, Buchanan, & Murata, 1994; Rosenbaum & Jorgensen, 1992; Schutz, Weigelt, Odekerken, Klein-Soetebier, & Schack, 2011; Short & Cauraugh, 1997), and the spatial paths of arm movements during reaching (Jax & Rosenbaum, 2007, 2009) and object use (Sorensen, Ingvaldsen, & Whiting, 2001).

Despite the relative prevalence of data demonstrating the effects of recent action history, also known as action hysteresis, the mechanics of the underpinning processes remain poorly understood. The most common interpretations suggest that computational gains underpin action hysteresis (Meulenbroek, Rosenbaum, Thomassen, & Schomaker, 1993; Rosenbaum, Chapman, Weigelt, Weiss, & van der Wel, 2012; Rosenbaum, Cohen, Jax, Weiss, & van der Wel, 2007; Weiss & Wark, 2009). Rather than computing entirely new plans for every new action, this model suggests that the brain makes adjustments to old plans that define recent actions, and that this “plan-modification” mechanism is computationally economical (Rosenbaum et al., 2007). When this model is applied to hand choice, reuse of the specification ‘hand’ is hypothesized to confer a relative computational benefit. We refer to this hypothesis as the computational efficiency model of action hysteresis.

Recent behavioural and neural data support this model. Response times to initiate actions are reduced when the same hand is used (Valyear & Frey, 2014), and these effects parallel reduced fMRI activity levels in brain areas that are important for action planning (Valyear & Frey, 2015). Both results are consistent with more efficient processing (Grill-Spector, Henson, & Martin, 2006; Henson, 2003; Wiggs & Martin, 1998). As a limitation, however, this prior work does not involve free choice about which hand to use to perform actions; hand use is instructed. The results may reflect more efficient action planning, specifically, and not extend to the processes that underpin hand choice.

The current study addresses this limitation and provides a new and critical test of the computational efficiency model of action hysteresis. No prior work has tested both hand choice and response times (RTs). This is nontrivial. If the computational efficiency model accounts for hand-choice hysteresis—the tendency to more often choose to use the hand that was used recently—then repeated hand use should result in reduced RTs to initiate actions, and the strengths of these effects should positively correlate. Individuals who show strong effects of history on hand choice should also show strong effects of history on response times. The current investigation provides the first test of these predictions.

Participants reach to contact visible targets using either hand. Targets are presented on either side of the participant’s midline, arranged in a semicircular array (see Fig. 1a). Hand choice is quantified as the point in target space where participants are equally likely to use either hand—the point of subjective equality (PSE)—computed separately according to whether previous trials (*t* − 1) involved the use of the left or right hand, named left-prime and right-prime conditions, respectively. If recent hand-use history influences hand choice, PSEs will differ depending on which hand was used in the previous trial. At the same time, if hysteresis reflects computational gains, repeated hand use should confer reduced RTs. Finally, if these two effects, hand-choice and RT hysteresis, reflect common underlying causes, as the computational efficiency model predicts, their strengths within participants should positively relate.

**Fig. 1:**
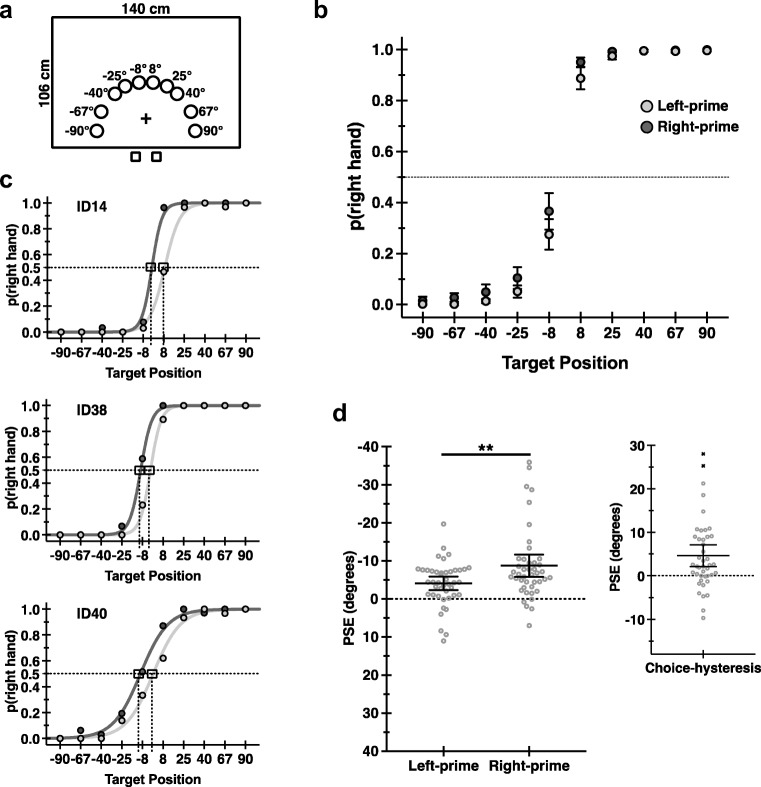
Choice-hysteresis as point of subjective equality (PSE) values. **a** Participants reach to contact targets at 10 positions. Squares represent the start positions of each hand. The “+” represents fixation. **b** Group mean proportions of right-hand use per target position per left-prime (light grey) and right-prime (dark grey) conditions are shown. Error bars indicate 95% confidence intervals. **c** Data from three participants illustrate individual-level fits of probability functions used to estimate PSE values per left-prime and right-prime conditions. Boxes drawn on curves show PSEs. **d** PSE data are shown as a function of left-prime and right-prime conditions (left), and as difference scores (left-prime − right-prime; right). Solid lines indicate group means with 95% confidence intervals, and open (light grey) circles show individual scores. Xs indicate outliers, shown for descriptive purposes, excluded from statistical analyses. ** indicates significance at *p* < .01

## Method

### Participants

Sixty individuals (43 female, mean age = 20.8 ± 4.2 years, age range: 18–51 years) from Bangor University participated in the experiment. All participants had normal or corrected-to-normal vision and provided informed consent in accordance with the Bangor University School of Psychology Ethics Board. A modified version of the Waterloo Handedness Inventory (Steenhuis & Bryden, 1989; scores range from −30 to +30) identified 51 participants as right-handers (mean score = 22.8 ± 6.2, range: 6–30; 38 female), and seven as left-handers (mean score = −13.4 ± −8.8, range: −1 to −24; five female). The experiment took approximately 1 hour to complete, and participants received course credits for their participation.

### Experimental setup and materials

Participants were seated at a 140-cm × 106-cm table, centred with respect to their midsagittal plane. The height of the table and chair was 81 cm and 65 cm, respectively. The table had a clear glass surface, and targets and the fixation point were projected onto the surface of the table using an upward-facing projector system. At the start of each trial, two start keys were held depressed with the index fingers of either hand. Start keys were fixed to the leading edge of the table, spaced 19.5 cm on centre. Targets were 4-cm-diameter circles projected onto the surface of the table at 10 positions relative to midline: −90, −67, −40, −25, −8, 8, 25, 40, 67, and 90 degrees. The target configuration approximates that used by Oliveira, Diedrichsen, Verstynen, Duque, and Ivry (2010). The average distance between targets and start keys was 40 cm. Participants could reach all targets comfortably with either hand. A fixation cross (4 cm × 4cm) was shown centrally, 25 cm from the leading edge of the table. The experiment was controlled using E-Prime Version 2.0.10.356 (Psychology Software Tools, Inc.).

### Procedure

Trials began with participants in the start position, holding down each of the start keys. Participants were instructed to fixate the central fixation cross. When the participant was ready, the experimenter initiated the trial. First, a 400-ms-duration tone was played to alert participants that the trial had started. This was followed by a variable delay (200/400/600/800 ms, randomly ordered). Next, for single-target conditions, a target appeared at one of the 10 positions of the target array (see Fig. 1a). Participants were instructed to reach to contact the target with the index finger of one hand, as quickly and accurately as possible. They were also told that they may move their eyes freely, and target onset was coincident with the removal of the fixation cross. Targets were made visible for 600 ms. Participants were instructed to use either hand to complete the task, and that one hand should remain holding the start key depressed. The next trial began as soon as the participant had returned to the start keys, and was fixating the central fixation cross. If participants erroneously moved both hands, the experimenter reminded them to only move one hand during single-target conditions.

There were two other kinds of conditions: two-target and fixation-catch trials. Two-target conditions involved the simultaneous presentation of two targets, presented at two of the 10 positions of the target array. Participants were instructed to use both hands to contact targets, and to attempt to move each hand together, at the same time. These trials were included to minimize the likelihood that participants would always use of the same hand for single-target conditions. Fixation-catch trials involved the presentation of a single target at fixation. Here, participants were instructed to use both hands to contact the target, and they were again told to move each hand together, synchronously. These trials were included to reinforce the likelihood that fixation would be maintained during the start of each trial, and to again minimize the likelihood that participants would always use of the same hand for single-target conditions.

Following initial instructions, participants completed a short block of 24 practice trials. All possible target locations were presented twice, and the practice trials included two two-target, and two fixation-catch trials. Feedback about whether responses for two-target and fixation-catch trials were correct was provided. The rest of the experiment was organized as six blocks of 145 trials. A custom MATLAB (R2011b) script was used to create trial sequences whereby trial (*t*) history (*t* − 1) is balanced according to condition, and target position for single-target conditions. Thus, each experimental block comprised 120 single-target trials, 12 per target position, and 24 two-target and fixation-catch trials, counterbalanced for *t* − 1 trial history. A unique trial sequence was generated per block. Data from practice trials, two-target and fixation-catch conditions, and the first trial of each block were excluded from analyses.

After all trials were completed, participants completed (1) the Waterloo Handedness Inventory, and (2) were asked if they ‘used a specific strategy, or rule’ to decide which hand to use. Left-handers and right-handers, strategy and nonstrategy users were defined as distinct groups. Questionnaires are provided in Supplemental Materials.

### Dependent measures and analyses

Outliers were defined as ± 2.5 standard deviations from the group mean, per statistical test, and removed from further analyses. Results from nonoutlier-removed analyses are reported in the Supplemental Materials.

All results are considered significant at *p* < .05. Where appropriate, Bonferroni correction was applied to post hoc follow-ups, with a corrected *p* < .05 taken as significant.

#### Hand choice

Hand choice was coded online by the experimenter, and confirmed off-line with button-release data. For each participant, a psychometric function (McKee, Klein, & Teller, 1985) was computed according to their hand choice behaviour (on single-target conditions) per target location, and the theoretical point in space where the participant was equally likely to use either hand—the point of subjective equality (PSE)—was determined. Specifically, PSE values are estimated by fitting a general linear model to each participant’s hand choice data. The model contains target positions and a constant term, and uses a logit link function to estimate the binomial distribution of hand choice responses (1 = right | 0 = left). Model coefficients are evaluated at 1,000 linearly spaced points between the outermost values of the target array (i.e. ± 90 degrees), and the value closest to a 0.50 probability estimate is defined as the PSE. The model was fitted separately per individual, per left-prime and right-prime conditions. The quality of each model fit was evaluated by correlating observed hand choice data per target location with the corresponding values estimated by the model, and the resultant *R*^2^ values were examined. Resultant PSEs per left-prime versus right-prime conditions were compared using a paired-samples *t* test.

Two additional analyses were performed. Hand-choice data expressed as proportions of right-hand use were first arcsine transformed, calculated as the arcsine square root of the proportions. The arcsine transformation stretches the upper and lower ends of the data. This makes the distributions more symmetrical and reduces problems with violations of the assumption of normality. The transformed proportions were then tested using two repeated-measures (RM) ANOVAs: (1) History (two levels: left-prime, right-prime) × Target Eccentricity (five levels: ± 90, 67, 40, 25, 8); (2) History (two levels: left-prime, right-prime) × Target Position (two levels: PSE, extreme).

#### Response times

Response times (RTs; i.e. time-to-action onsets) are defined as the time from target onset to the release of the start keys.

Two RM-ANOVAs were used to evaluate RT data: (1) History (two levels: switch, repeat) × Hand (two levels: left hand, right hand); (2) History (two levels: switch, repeat) × Target Position (two levels: PSE, extreme).

#### Choice hysteresis and RT hysteresis

A simple linear-regression analysis was used to test for a significant relationship between history effects on hand choice and RTs. Choice hysteresis was defined as the difference values between left-prime PSEs minus right-prime PSEs, and RT hysteresis was defined as the difference values between switch RTs minus repeat RTs. Positive values correspond to predicted directions of hysteresis.

## Results

Data reported include right-handers without strategy use (*N* = 43). All statistical outcomes are provided in Table 1. Results from the complete data set, including left-handers (*N* = 7) and right-handers who report strategy use (*N* = 8), are provided in the Supplemental Materials.

**Table 1:** Statistical outcomes, right-handers no-strategy (*N* = 43)

(a) Hand choice	
A-1: History	A-2: History by target eccentricity
DV: PSE values	DV: Arcsine transformed p(RHU)
Test: Paired-samples *t* test	Test: RM-ANOVA History (2) × Target Position (5)
*N* = 40, outlier removed	*N* = 40, outlier removed
Left-prime − right-prime: *t*(40) = 3.48, ***p*< .005**	Main effect: History: *F*(1, 39) = 9.88, ***p*< .005**
	Main effect: Target position: *F*(4, 36) = 10.53, *p*< .001*
	Interaction: *F*(4, 36) = 5.88, *p*< .001*
	*Greenhouse–Geisser applied
A-3: History by (PSE/Extreme) target position	
DV: Arcsine transformed p(RHU)	
Test: RM-ANOVA History (2) × Target Position (2)	
*N* = 42, outlier removed	
Main effect: History: *F*(1, 41) = 15.8, ***p*< .001**	
Main effect: Target location: *F*(1, 41) = 2.68, *p* = .11	
Interaction: *F*(1, 41) = 18.4, ***p*< .001**	
(b) Response times	
B-1: History by hand	B-2: History by (PSE/Extreme) target position
DV: RTs	DV: RTs
Test: RM-ANOVA Hand (2) × History (2)	Test: RM-ANOVA History (2) × Target Position (2)
*N* = 42, outlier removed	*N* = 43, no outliers detected
Main effect: Hand: *F*(1, 41) = 0.59, *p* = .45	Main effect: History: *F*(1, 42) = 4.96, ***p*< .05**
Main effect: History: *F*(1, 41) = 41.0, ***p*< .001**	Main effect: Target location: *F*(1, 42) = 154.2, ***p*< .001**
Interaction: *F*(1, 41) = 2.31, *p* = .14	Interaction: *F*(1, 42) = 0.53, *p* = .47
(c) Choice hysteresis and RT hysteresis	
DV: PSE and RTs	
Test: Linear regression	
*N* = 40, outlier removed	
ANOVA: *F*(1, 38) = 4.42, ***p*< .05**; *R*^2^ = .11	
Pearson correlation = 0.32	
Cook’s distance, max = 0.44	
Durbin–Watson = 1.77	

Bolded text highlight tests that reach significance

Participants made few errors. These include a total of 201 trials involving early responses (prior to stimulus onset), and 287 trials involving multiple start-key releases, comprising 0.8% and 1.1% of the single-target data, respectively. For the majority of multiple key-release errors (209/287), the hand that is used to reach to contact targets is unambiguous (confirmed via video recordings), and thus these data are retained for hand-choice analyses. Otherwise, all error trials, and those trials that immediately follow errors, are excluded from analyses.

Bimanual catch trials were also performed with few errors: 201 early responses, comprising 3.2% of these data.

### Hand choice

Hand choice varies as a function of target position (see Fig. 1b). Responses to more lateralized (± 90°/67°/40°) targets typically involve the use of the ipsilateral hand. Our curve-fitting methods used to estimate individual-level PSE values (see Hand Choice section) provide excellent fits to the data, qualified by correlating observed hand-choice data at each target position with the model estimates. The average coefficients expressed as *R*^2^ values are 0.993 and 0.998 for right-prime and left-prime data, respectively. Figure 1c shows three examples.

Analyses of PSE data reveal significant effects of history on hand choice (see Fig. 1d; Table 1, A-1). PSEs are decreased for right-prime versus left-prime conditions, shifted leftward in target space. Participants are more likely to use their right hand to reach to targets in left hemispace on a given trial (*t*) when the previous trial (*t* − 1) involves the use of the right hand. These results are consistent with predicted effects of history on hand choice. We define these effects as choice hysteresis.

**Fig. 2:**
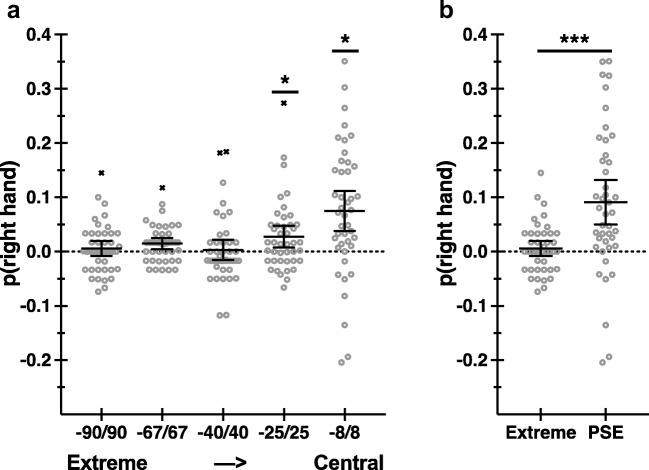
Choice hysteresis as proportions of right-hand use. **a** Proportions of right-hand use are shown as difference scores (right-prime − left-prime) as a function of target eccentricity. Positive values are consistent with predicted effects of history (choice hysteresis). Solid lines indicate group means with 95% confidence intervals, and open circles show individual scores. Xs indicate outliers, shown for descriptive purposes only. Untransformed data are shown, for ease of interpretation. Statistical analyses are performed on arcsine transformed data (see Hand Choice section). * indicates significant post hoc pairwise comparisons at *p* < .05, Bonferroni corrected. **b** Same as **a**, shown for extreme and PSE target positions. *** indicates significance at *p* < .001

Complementary analyses of hand-choice data as arcsine transformed proportions yield consistent results and, moreover, demonstrate a gradient of sensitivity to recent hand-use history as a function of target eccentricity (see Fig. 2a; Table 1, A-2). Specifically, the effects of recent history on hand choice are significant for targets near the midline, at ±25° and 8°, and are statistically unreliable for more lateralized targets, at ±90°/67°/40°. Notably, these analyses are independent from our curve-fitting methods and PSE estimates.

**Fig. 3:**
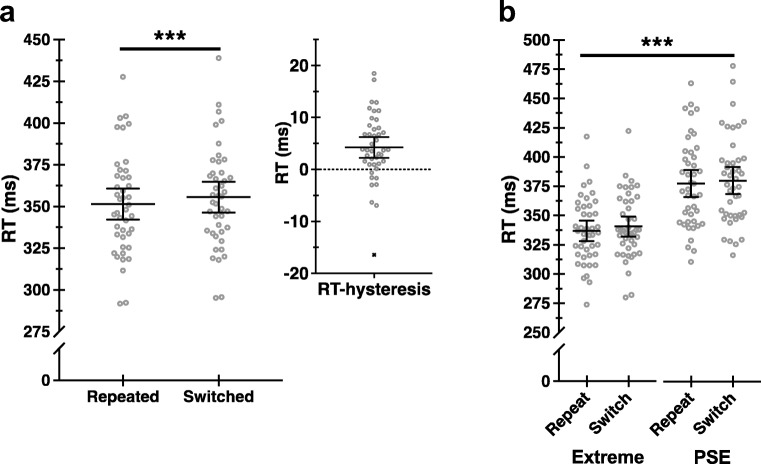
RT hysteresis. **a** Response-time data are plotted as a function of repeat and switch conditions (left), and as difference scores (switch − repeat) (right). Solid lines indicate group means with 95% confidence intervals, and open circles show individual scores. Xs denote outliers, shown for descriptive purposes only. **b** Same as **a**, shown for extreme and PSE target positions. *** indicates significance at *p* < .001

Finally, we perform a similar analysis, but instead quantify hand choice as arcsine transformed proportions per left-prime and right-prime conditions for responses to targets that bound the PSE, defined per individual, and compare these data with responses to targets at Extreme (±90°) lateral positions. The effects of recent history are significant for responses to PSE-bound targets (see Fig. 2b; Table 1, A-3). This analysis is performed merely for comparison with our data shown in Fig. 2a, involving all target eccentricities, and to parallel a complementary analysis of RT data, reported below (see Fig. 3b).

### Response times

Participants are significantly faster to respond when the same hand is used successively, for repeat compared with switch conditions (see Fig. 3a; Table 1, B-1). These results are consistent with the predicted effects history on RTs. We define these effects as RT hysteresis. No significant main effect of Hand nor Hand × History interaction are identified (see Table 1, B-1).

Separate analyses reveal that RTs are significantly prolonged for reaches to targets that bound the PSE compared to those at extreme lateral positions (see Fig. 3b; Table 1, B-2). These analyses are motivated by results from Oliveira et al. (2010), who, using a similar target configuration, reveal prolonged RTs for reaches to PSE-bound versus extreme targets. Our results are consistent with their findings. Further, Oliveira et al. (2010) include a control task involving instructed hand use and demonstrate that prolonged RTs to PSE-bound targets are specific to the free-choice task. This suggests that these effects reflect graded decision costs as a function of target position—decision times are prolonged where the biomechanical and energetic costs of using either hand are comparable. We interpret our data similarly. The differences in RTs for PSE − extreme targets are interpreted as the added time required to make a choice when the intermanual action costs are comparable.

No significant interaction between target position and history is identified (see Table 1, B-2). A significant main effect of history reflects RT hysteresis, as reported above (see Fig. 3a; Table 1, B-1).

### Choice hysteresis and RT hysteresis

**Fig. 4:**
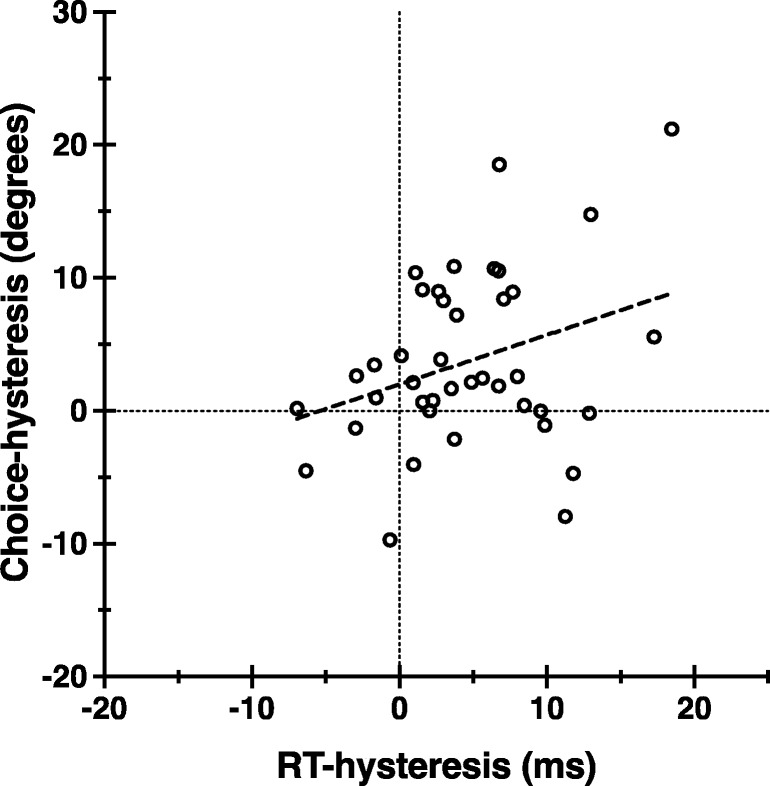
The relationship between choice hysteresis and RT hysteresis. Individual-level choice hysteresis (left-prime PSE − right-prime PSE) data are plotted as a function of individual-level RT hysteresis (switch RT − repeat RT) data. Linear regression indicates a significant positive relationship at *p* < .05. Outliers are excluded (see Figs. 1d and 3a, respectively)

Linear regression reveals a significant positive relationship between choice hysteresis and RT hysteresis (see Fig. 4; Table 1C). Those individuals who show a strong influence of prior hand-use history on hand choice also tend to show a strong influence of prior hand-use history on RTs. These findings are consistent with the computational efficiency model of action hysteresis: Both results—Choice hysteresis and RT hysteresis—can be interpreted as improved processing efficiency when successive actions involve the use of the same hand. History influences hand choice and confers a response-time advantage/cost.

## Discussion

The current data reveal that recent action history influences hand choice and demonstrate that these effects parallel differences in response times. Participants are more likely to choose the same hand that was used recently, in particular when the biomechanical and energetic costs of performing actions with either hand are similar, and repeated choices confer response-time gains to initiate actions. The effects of both choice hysteresis and RT hysteresis are small but reliable, and positively correlate within individuals. A response-time advantage for repeated choices is consistent with the computational efficiency model—when the same action choices are made repeatedly, the underlying processes complete more efficiently. We discuss our results within the framework of this model, and speculate about the possible underlying mechanisms in neural terms.

By linking the effects of recent history on action choices and response times, the current findings provide new support for a computational efficiency interpretation of action hysteresis. Consistent with our data, previous findings demonstrate the influence of recent action history on hand choice (Rostoft et al., 2002; Schweighofer et al., 2015; Weiss & Wark, 2009), but critically, in this prior work, response times were not also tested. Conversely, other data indicate that response times are reduced when the same hand is used (Valyear & Frey, 2014, 2015), but in this work, hand choice was not tested. Here, we show that recent action history affects both current hand choice and response times to initiate actions, and reveal a statistically reliable relationship between them. Choice hysteresis accompanied by reduced response times to initiate actions provides new support for the computational efficiency model.

Action hysteresis as a computational efficiency phenomenon can be understood within the framework of action selection models that emphasize the importance of balancing estimated costs and benefits. For example, according to the model developed by Shadmehr et al. (2016), the brain computes a “utility” estimate of possible actions that reflects a balance between predicted energetic costs and reward values, and the results of these computations determine both which actions to perform—action selection—and how to move. Energetic costs are estimated directly from the metabolic energy needed to produce possible actions, and action utility is computed as the temporally discounted sum of these costs and the estimated reward values associated with those actions. Their model accounts for various experimental data, including both the choices and movement speeds made during reaching. Within this framework, action hysteresis can be understood as a consequence of reduced energetic costs. Here, the computational savings presumably map to metabolic processes within the central nervous system, as supported by fMRI data (see below), and must offset the costs of otherwise suboptimal movements (Shadmehr & Krakauer, 2008, p. 379).

The current data are consistent with this interpretation. In our task, the biomechanical and energetic costs associated with the use of either hand differ according to target position. For targets at extreme lateral positions, the ipsilateral hand is strongly favoured. According to bounded accumulation models of decision-making (Gold & Shadlen, 2007; Kiani, Hanks, & Shadlen, 2008; Ratcliff, Cherian, & Segraves, 2003), decisions are made when the activity of neurons representing the relevant decision variables reach a critical threshold. Since biomechanical factors constitute relevant decision variables for hand choice, these factors are expected to influence accumulation-to-threshold rates. A strong bias for selecting the ipsilateral hand at extreme lateral target positions can be interpreted as faster accumulation rates to reach selection thresholds for neurons that represent actions with the ipsilateral hand. Conversely, the biomechanical costs of reaching to targets near the midline are similar for actions with either hand, and thus the accumulation rates to reach selection thresholds will also be similar. Our RT data showing prolonged responses for reaching to targets near the midline and PSE support this view. Moreover, our choice-hysteresis results follow this gradient. When intermanual action costs, and, consequently, rise-to-threshold rates are comparable, as for targets near the midline and PSE, hysteresis has a significant influence on hand choice. Conversely, when intermanual action costs are highly asymmetrical, as for extreme lateralized targets, choice hysteresis is negligible. Here, the intermanual differences in accumulation rates outweigh the processing gains related to repeated hand use. In other words, when the biomechanical costs of repeating recent action choices are high, choice hysteresis is minimal (see also Cohen & Rosenbaum, 2011).

Although speculative, we suggest that our results reflect the recycling of recently specified motor parameters that persist within the cortical sensorimotor control system. Repeated hand use is associated with reduced fMRI responses within brain areas in the posterior parietal cortex that are important for the planning and control of reaching actions (Valyear & Frey, 2015), and these effects are consistent with decreases in neural-metabolic processing costs (Grill-Spector et al., 2006). Other data suggest that action selection involves competition between concurrently active neural populations within sensorimotor areas (in posterior parietal and premotor cortices) that specify the spatiotemporal parameters of possible actions (Cisek, 2007; Cisek & Pastor-Bernier, 2014; Gold & Shadlen, 2000; Hanks, Ditterich, & Shadlen, 2006). Applied to our data, choice and RT hysteresis can be understood as changes in the baseline levels of activity within competing neural populations that encode hand-specific action plans as a consequence of residual encoding from recently specified actions.

Prior results are consistent with a competitive process underlying hand choice and involving posterior parietal brain areas that are important for reach control (Oliveira et al., 2010), and trial history has been shown to influence both the RTs to initiate saccadic eye movements and the baseline activity levels of neurons responsible for controlling those movements (Fecteau & Munoz, 2003). Also, hysteresis reflected in the spatial paths of arm movements diminishes rapidly with time between successive movements, a results that is consistent with the hypothesis that action hysteresis reflects the reuse of residual parameters within the sensorimotor system (Jax & Rosenbaum, 2009).

Despite this evidence, we recognize that our interpretation is speculative, and that not all data support this view. Specifically, rather than sensorimotor in nature, Dixon et al. (2012) demonstrate effects of recent action history on how the hand is shaped to grasp objects that are better explained according to episodic memory representations. Their hysteresis results are coupled to visual object properties, sensitive to contextual similarity, and resistant to motor interference from intermediate responses involving nonrepeated grasps. Other data demonstrate grasp hysteresis that reflects object-centred rather than body-centered representations (Weigelt, Cohen, & Rosenbaum, 2007), and that action tasks involving high-level planning (and hysteresis) influence declarative memory recall, suggesting that action planning and verbal working memory share cognitive resources (Weigelt, Rosenbaum, Huelshorst, & Schack, 2009). It is also worth noting that other studies reveal history effects that transfer between hands, and thus reflect the influence of abstract motor representations (Dixon et al., 2012; van der Wel, Fleckenstein, Jax, & Rosenbaum, 2007). These data contrast with our sensorimotor-level interpretation of action hysteresis and illustrate opportunities for future research. It may be, for example, that distinct variations of action hysteresis, operating at different levels of processing, are possible, and co-occur.

Unfortunately, the current design is not appropriately suited to address possible history effects that may accrue beyond trial *t* − 1. Redefining our conditions to include *t* ≥ 1 trial history will result in too few trials per target position to reliably estimate PSE values for left-prime and right-prime conditions. Previous evidence suggests that multiple repetitions can lead to cumulative effects of history on action planning and performance (Song & Nakayama, 2007; Whitwell & Goodale, 2009; Whitwell, Lambert, & Goodale, 2008). Whether similar cumulative hysteresis effects also emerge for hand choice will require future experiments, beyond the scope of the current study.

Our findings demonstrate that when participants are free to choose which hand to use to reach to contact targets repeated choices result in reliably shorter responses times, and when the action costs between hands are similar, these choices are biased in favour of which hand was used recently. These results provide new support for a computation efficiency interpretation of action hysteresis and are interpretable within the context of action selection models that emphasize the importance of balancing estimated gains and costs. We speculate that parallel choice and RT hysteresis reflect a common underlying mechanism involving the respecification of residual sensorimotor parameters. Altogether, the current data significantly advance our knowledge of action hysteresis and provide valuable points of comparison for future research.

## Electronic supplementary material

ESM 1 (PDF 432 kb)
