# Supplementary material for: Now and then: Hand choice is influenced by recent action history
Source: Psychon Bull Rev. 2018 Jul 23;26(1):305–14. doi: 10.3758/s13423-018-1510-1 (PMC6424939; doi:10.3758/s13423-018-1510-1)
Supplement: Supplementary file 1 — (PDF 432 kb) [file 13423_2018_1510_MOESM1_ESM.pdf]

Supplemental Materials

For each test reported in the main manuscript, Tables S1-S6, below, provide the results from non-outlier-removed analyses, and from analyses of the complete dataset (N = 58) including Left-handers (N = 7) and Right-handers who report strategy use (N = 8).

S1. Strategy Use

A subset of Right-handers (8/51) were identified as Strategy-users. No Left-handers reported the use of a strategy. When strategy-use was indicated, (see Strategy Questionnaire, below), the experimenter reviewed the description provided. Among the few participants that reported the use of a strategy, a common principle was identified – i.e. if stimuli were presented on the left, they used their left hand, and if stimuli were presented on the right, they used their right hand.

Notably, results of the PSE analyses from the full dataset (Table S1-1/2) indicate that Right-handers Strategy and No-strategy groups do not differ ($p > 0.99$, for outlier removed and non-removed data); the Strategy group shows a similar right-hand selection bias, despite the fact that they report using a rule-based strategy to decide which hand to use.

S2. Hand Preference

Using a cut-off < 0 = Left-handed, the modified Waterloo scale (provided below) identified 7 participants as Left-handed (mean score = -13.4 +/- -8.8, range = -1 to -24; 5 female).

Two sets of results suggest that our task is sensitive to hand preference, as defined by the modified Waterloo scale.

First, analyses of PSE data using a mixed ANOVA reveal a significant main effect of Group (Table S1-2; Figure S1). Post-hoc follow-ups reveal significant differences between Left-handers (mean = 5.3°, SEM +/- 5.3) and Right-handers No-strategy (mean = -5.8°, SEM +/- 1.2) ($p < 0.005$) and Right-handers Strategy (mean = -5.7°, SEM +/- 2.6) ($p < 0.05$). These results are consistent with a significant effect of hand preference on hand choice, with Left-handers showing more positive PSEs.

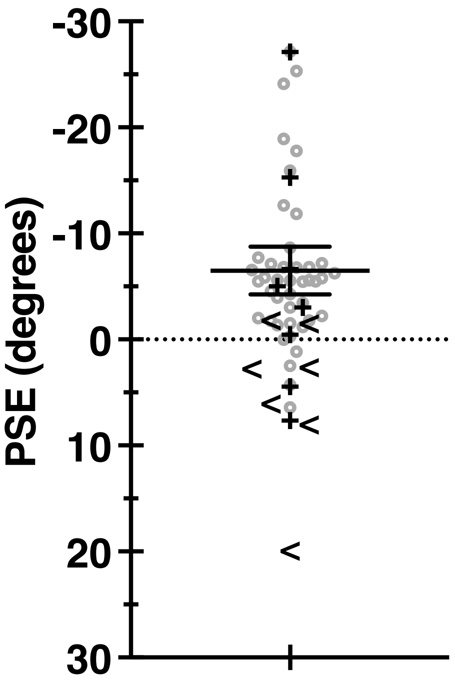

Figure S1. PSE values per group. Individual-level PSE values are plotted. Right-handers who do not report strategy use are shown as open (light grey) circles, and the solid lines indicate the mean value and 95% confidence intervals for this group (N = 43). PSE values of Left-handers (N = 7) are shown as "<", and Right-handers (N = 8) who report the use of a strategy are indicated as "+". No Left-handers report the use of a strategy. As a group, Left-handers exhibit a mean PSE that is significantly more positive than both groups of Right-handers (see Table S1-1/2).

Second, additional analyses using simple linear regression reveal a significant negative relationship between Waterloo scores and PSEs (Table S7; Figure S2).

Together, these results are consistent with a host of previous data showing that manual tasks involving free hand choice are sensitive to hand preference (e.g., Bryden et al., 1994; Bishop et al., 1996; Stins et al., 2001).

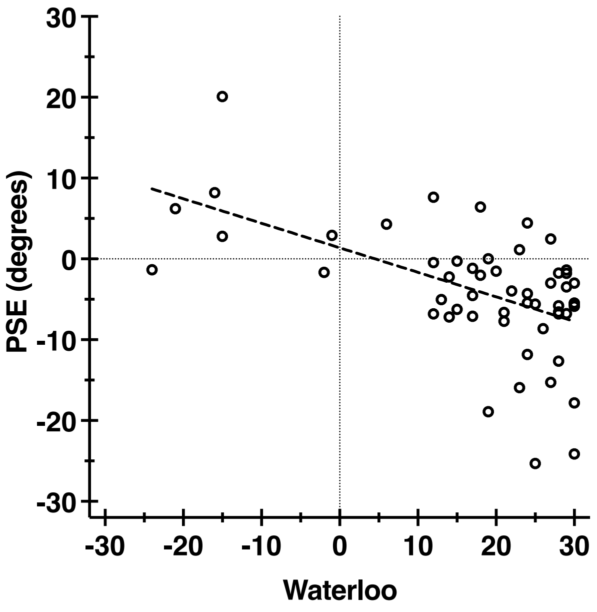

Figure S2. The relationship between PSE and Waterloo scores. Individual-level PSE values are plotted as a function of individual-level modified Waterloo scores (Steenhuis and Bryden, 1989). Modified Waterloo scores range from -30 to +30, indicative of strongly left- versus right-handedness, respectively. Linear regression indicates a significant negative relationship at $p < 0.05$ (see Table S7-2). Outliers in PSE data are excluded.

References

Bishop DV, Ross VA, Daniels MS, Bright P (1996) The measurement of hand preference: a validation study comparing three groups of right-handers. Br J Psychol 87 (Pt 2):269-285.

Bryden MP, Singh M, Steenhuis RE, Clarkson KL (1994) A behavioral measure of hand preference as opposed to hand skill. Neuropsychologia 32:991-999.

Steenhuis RE, Bryden MP (1989) Different dimensions of hand preference that relate to skilled and unskilled activities. Cortex 25:289-304.

Stins JF, Kadar EE, Costall A (2001) A kinematic analysis of hand selection in a reaching task. Laterality 6:347-367.

Table S1. Hand choice: PSE values: History

(S1-1) Full dataset (N = 58): mixed ANOVA History (2) x Group (3)

Main effect: History: F (1, 55) = 4.34, p < 0.05
Main effect: Group: F (2, 55) = 7.54, p < 0.005
Interaction: History x Group: F (2, 55) = 0.84, p = 0.44

Box's test:

Box's M = 10.35, F (6, 2508) = 1.52, p = 0.17

Levene's test:

Left-prime: F (2, 55) = 0.57, p = 0.57
Right-prime: F (2, 55) = 0.81, p = 0.45

Tests of normality:

Choice-hysteresis: Shapiro-Wilk (58) = 0.93, p < 0.005

(S1-2) Outlier removed (N = 56): mixed ANOVA History (2) x Group (3)

Main effect: History: F (1, 53) = 4.72, p < 0.05
Main effect: Group: F (2, 53) = 7.41, p < 0.005
Interaction: History x Group: F (2, 53) = 0.56, p = 0.58

Box's test:

Box's M = 8.79, F (6, 2533) = 1.29, p = 0.27

Levene's test:

Left-prime: F (2, 53) = 0.47, p = 0.63
Right-prime: F (2, 53) = 1.63, p = 0.21

Tests of normality:

Choice-hysteresis: Shapiro-Wilk (56) = 0.97, p = 0.11

(S1-3) Right-handers No-strategy (N = 43): paired samples t-test History (2)

Left-prime – Right-prime = t (42) = 3.78, p < 0.005

Tests of normality:

Choice-hysteresis: Shapiro-Wilk (43) = 0.92, p < 0.01

(S1-4) Right-handers No-strategy, outlier removed (N = 41): paired samples t-test History (2)

Left-prime – Right-prime = t (40) = 3.48, p < 0.005

Tests of normality:

Choice-hysteresis: Shapiro-Wilk (41) = 0.96, p = 0.21

Table S2. Hand choice: arcsine transformed p(RHU): History by Target Eccentricity

(S2-1) Full dataset (N = 58): mixed ANOVA History (2) x Target Location (5) x Group (3)

Main effect: History: F (1, 54) = 2.76, p = 0.10
Main effect: Target Eccentricity: F (1.11, 60.04) = 1.54, p = 0.22*
Main effect: Group: F (2, 54) = 6.65, p < 0.005
Interaction: History x Target Eccentricity: F (1.87, 101.03) = 0.70, p = 0.49*
Interaction: History x Group: F (2, 54) = 1.03, p = 0.37
Interaction: Target Eccentricity x Group: F (2.24, 60.04) = 8.98, p < 0.001*
Interaction: History x Target Eccentricity x Group: F (3.74, 101.03) = 2.43, p = 0.057*

**Greenhouse-Giesser applied*

Levene's test:

Left-prime: -90/+90 (Extreme): F (2, 54) = 0.97, p = 0.39
Left-prime: -67/+67: F (2, 54) = 1.39, p = 0.26
Left-prime: -25/+25: F (2, 54) = 0.72, p = 0.49
Left-prime: -25/+25: F (2, 54) = 3.35, p = 0.043
Left-prime: -8/+8 (Central): F (2, 54) = 1.17, p = 0.32
Right-prime: -90/+90 (Extreme): F (2, 54) = 0.99, p = 0.38
Right-prime: -67/+67: F (2, 54) = 1.12, p = 0.33
Right-prime: -25/+25: F (2, 54) = 1.16, p = 0.32
Right-prime: -25/+25: F (2, 54) = 0.20, p = 0.82
Right-prime: -8/+8 (Central): F (2, 54) = 4.15, p = 0.02

Mauchly's test of sphericity:

Eccentricity: Mauchly's W < 0.01, p < 0.001
History x Target Eccentricity: Mauchly's W = 0.14, p < 0.001

Tests of normality:

Right-Prime – Left-prime: -90/+90 (Extreme): Shapiro-Wilk (57) = 0.98, p = 0.04
Right-Prime – Left-prime: -67/+67: Shapiro-Wilk (57) = 0.96, p = 0.084
Right-Prime – Left-prime: -25/+25: Shapiro-Wilk (57) = 0.88, p < 0.001
Right-Prime – Left-prime: -25/+25: Shapiro-Wilk (57) = 0.90, p < 0.001
Right-Prime – Left-prime: -8/+8 (Central): Shapiro-Wilk (57) = 0.99, p = 0.69

(S2-2) Outlier removed (N = 55): mixed ANOVA History (2) x Target Location (5) x Group (3)

Main effect: History: F (1, 51) = 2.70, p = 0.11
Main effect: Target Eccentricity: F (1.10, 56.00) = 1.26, p = 0.27*
Main effect: Group: F (2, 51) = 6.83, p < 0.005
Interaction: History x Target Eccentricity: F (1.87, 88.32) = 1.73, p = 0.48*
Interaction: History x Group: F (2, 51) = 0.60, p = 0.55
Interaction: Target Eccentricity x Group: F (2.12, 56.00) = 8.49, p < 0.001*
Interaction: History x Target Eccentricity x Group: F (3.46, 88.32) = 2.36, p = 0.068*

**Greenhouse-Giesser applied*

Levene's test:

Left-prime: -90/+90 (Extreme): F (2, 51) = 0.97, p = 0.32
Left-prime: -67/+67: F (2, 51) = 1.39, p = 0.23
Left-prime: -25/+25: F (2, 51) = 0.72, p = 0.37
Left-prime: -25/+25: F (2, 51) = 3.35, p = 0.032
Left-prime: -8/+8 (Central): F (2, 51) = 1.17, p = 0.30
Right-prime: -90/+90 (Extreme): F (2, 51) = 0.99, p = 0.30
Right-prime: -67/+67: F (2, 51) = 1.12, p = 0.006
Right-prime: -25/+25: F (2, 51) = 1.16, p = 0.20
Right-prime: -25/+25: F (2, 51) = 0.20, p = 0.89
Right-prime: -8/+8 (Central): F (2, 51) = 4.15, p = 0.006

Mauchly's test of sphericity:

Eccentricity: Mauchly's W < 0.01, p < 0.001
History x Target Eccentricity: Mauchly's W = 0.11, p < 0.001

Tests of normality:

Right-Prime – Left-prime: -90/+90 (Extreme): Shapiro-Wilk (54) = 0.98, p = 0.40
Right-Prime – Left-prime: -67/+67: Shapiro-Wilk (54) = 0.96, p = 0.22

Right-Prime – Left-prime: -25/+25: Shapiro-Wilk (54) = 0.88, p = 0.01
Right-Prime – Left-prime: -25/+25: Shapiro-Wilk (54) = 0.90, p = 0.083
Right-Prime – Left-prime: -8/+8 (Central): Shapiro-Wilk (54) = 0.99, p = 0.65

(S2-3) Right-handers No-strategy (N = 43): RM-ANOVA History (2) x Target Location (5)

Main effect: History: F (1, 42) = 13.63, p < 0.005
Main effect: Target Eccentricity: F (1.15, 48.10) = 42.45, p < 0.001*
Interaction: History x Target Eccentricity: F (1.83, 76.83) = 11.35, p < 0.001*
**Greenhouse-Giesser applied*

Mauchly's test of sphericity:
Eccentricity: Mauchly's W < 0.01, p < 0.001
History x Target Eccentricity: Mauchly's W = 0.13, p < 0.001

Tests of normality:
Right-Prime – Left-prime: -90/+90 (Extreme): Shapiro-Wilk (43) = 0.95, p = 0.07
Right-Prime – Left-prime: -67/+67: Shapiro-Wilk (43) = 0.93, p = 0.013
Right-Prime – Left-prime: -40/+40: Shapiro-Wilk (43) = 0.87, p < 0.001
Right-Prime – Left-prime: -25/+25: Shapiro-Wilk (43) = 0.86, p < 0.001
Right-Prime – Left-prime: -8/+8 (Central): Shapiro-Wilk (43) = 0.99, p = 0.90

(S2-4) Right-handers No-strategy, outlier removed (N = 40): RM-ANOVA History (2) x Target Location (5)

Main effect: History: F (1, 39) = 9.88, p < 0.005
Main effect: Target Eccentricity: F (1.13, 43.87) = 34.95, p < 0.001*
Interaction: History x Target Eccentricity: F (1.65, 64.28) = 10.43, p < 0.001*
**Greenhouse-Giesser applied*

Mauchly's test of sphericity:
Eccentricity: Mauchly's W < 0.01, p < 0.001
History x Target Eccentricity: Mauchly's W = 0.088, p < 0.001

Tests of normality:
Right-Prime – Left-prime: -90/+90 (Extreme): Shapiro-Wilk (40) = 0.97, p = 0.33
Right-Prime – Left-prime: -67/+67: Shapiro-Wilk (40) = 0.95, p = 0.062
Right-Prime – Left-prime: -40/+40: Shapiro-Wilk (40) = 0.91, p = 0.005
Right-Prime – Left-prime: -25/+25: Shapiro-Wilk (40) = 0.94, p = 0.037
Right-Prime – Left-prime: -8/+8 (Central): Shapiro-Wilk (40) = 0.98, p = 0.83

Table S3. Hand choice: arcsine transformed p(RHU): History by (PSE/Extreme) Target Position

(S3-1) Full dataset ($N = 58$): mixed ANOVA History (2) x Target Location (2) x Group (3)

Main effect: History: $F(1, 55) = 1.95$, $p = 0.17$
Main effect: Target Location: $F(1, 55) = 1.20$, $p = 0.28$
Main effect: Group: $F(2, 55) = 1.72$, $p = 0.19$
Interaction: History x Target Location: $F(1, 55) = 2.86$, $p = 0.10$
Interaction: History x Group: $F(2, 55) = 1.78$, $p = 0.18$
Interaction: Target Location x Group: $F(2, 55) = 1.69$, $p = 0.19$
Interaction: History x Target Location x Group: $F(2, 55) = 1.93$, $p = 0.16$

Box's test:

Box's $M = 50.79$, $F(2, 1017) = 1.92$, $p < 0.01$

Levene's test:

Left-prime Extreme: $F(2, 55) = 0.89$, $p = 0.42$
Left-prime PSE: $F(2, 55) = 1.59$, $p = 0.21$
Right-prime Extreme: $F(2, 55) = 0.99$, $p = 0.38$
Right-prime PSE: $F(2, 55) = 3.16$, $p = 0.05$

Tests of normality:

Choice-hysteresis: Extreme: Shapiro-Wilk $(58) = 0.96$, $p = 0.041$
Choice-hysteresis: PSE: Shapiro-Wilk $(58) = 0.96$, $p = 0.037$

(S3-2) Outlier removed ($N = 57$): mixed ANOVA History (2) x Target Location (2) x Group (3)

Main effect: History: $F(1, 54) = 2.00$, $p = 0.16$
Main effect: Target Location: $F(1, 54) = 1.1$, $p = 0.30$
Main effect: Group: $F(2, 54) = 2.47$, $p = 0.094$
Interaction: History x Target Location: $F(1, 54) = 2.79$, $p = 0.10$
Interaction: History x Group: $F(2, 54) = 1.68$, $p = 0.20$
Interaction: Target Location x Group: $F(2, 54) = 2.64$, $p = 0.08$
Interaction: History x Target Location x Group: $F(2, 54) = 1.72$, $p = 0.19$

Box's test:

Box's $M = 57.15$, $F(2, 1020) = 2.16$, $p < 0.005$

Levene's test:

Left-prime Extreme: $F(2, 54) = 0.94$, $p = 0.40$
Left-prime PSE: $F(2, 54) = 2.98$, $p = 0.06$
Right-prime Extreme: $F(2, 54) = 1.1$, $p = 0.34$
Right-prime PSE: $F(2, 54) = 3.55$, $p = 0.04$

Tests of normality:

Choice-hysteresis: Extreme: Shapiro-Wilk $(57) = 0.98$, $p = 0.64$
Choice-hysteresis: PSE: Shapiro-Wilk $(57) = 0.97$, $p = 0.15$

(S3-3) Right-handers No-strategy ($N = 43$): RM-ANOVA History (2) x Target Location (2)

Main effect: History: $F(1, 42) = 15.5$, $p < 0.001$
Main effect: Target Location: $F(1, 42) = 0.68$, $p = 0.42$
Interaction: History x Target Location: $F(1, 42) = 19.6$, $p < 0.001$

Tests of normality:

Choice-hysteresis: Extreme: Shapiro-Wilk $(43) = 0.95$, $p = 0.071$
Choice-hysteresis: PSE: Shapiro-Wilk $(43) = 0.95$, $p = 0.073$

(S3-4) Right-handers, No-strategy, outlier removed ($N = 42$): RM-ANOVA History (2) x Target Location (2)

Main effect: History: $F(1, 41) = 15.8$, $p < 0.001$
Main effect: Target Location: $F(1, 41) = 2.68$, $p = 0.11$
Interaction: History x Target Location: $F(1, 41) = 18.4$, $p < 0.001$

Tests of normality:

Choice-hysteresis: Extreme: Shapiro-Wilk $(42) = 0.97$, $p = 0.38$
Choice-hysteresis: PSE: Shapiro-Wilk $(42) = 0.97$, $p = 0.31$

Table S4. Response Times: History by Hand

(S4-1) Full dataset (N = 58): mixed ANOVA Hand (2) x History (2) x Group (3)

Main effect: Hand: F (1, 55) = 0.29 p = 0.59
Main effect: History: F (1, 55) = 14.6, p < 0.001
Main effect: Group: F (2, 55) = 1.57, p = 0.22
Interaction: Hand x History: F (1, 55) = 1.11, p = 0.30
Interaction: Hand x Group: F (2, 55) = 0.58, p = 0.57
Interaction: History x Group: F (2, 55) = 0.13, p = 0.88
Interaction: Hand x History x Group: F (2, 55) = 0.32, p = 0.73

Box's test:

Box's M = 15.57, F (20, 1017) = 0.58, p = 0.92

Levene's test:

Left Hand Repeat: F (2, 55) = 0.08, p = 0.92
Left Hand Switch: F (2, 55) = 0.52, p = 0.60
Right Hand Repeat: F (2, 55) = 0.42, p = 0.66
Right Hand Switch: F (2, 55) = 0.85, p = 0.43

Tests of normality:

RT-hysteresis: Shapiro-Wilk (58) = 0.98, p = 0.40

(S4-2) Outlier removed (N = 57): mixed ANOVA Hand (2) x History (2) x Group (3)

Main effect: Hand: F (1, 54) = 0.23 p = 0.63
Main effect: History: F (1, 54) = 18.4, p < 0.001
Main effect: Group: F (2, 54) = 1.46, p = 0.24
Interaction: Hand x History: F (1, 54) = 1.11, p = 0.30
Interaction: Hand x Group: F (2, 54) = 0.56, p = 0.57
Interaction: History x Group: F (2, 54) = 0.12, p = 0.88
Interaction: Hand x History x Group: F (2, 54) = 0.28, p = 0.75

Box's test:

Box's M = 14.94, F (20, 1020) = 0.56, p = 0.94

Levene's test:

Left Hand Repeat: F (2, 54) = 0.12, p = 0.89
Left Hand Switch: F (2, 54) = 0.53, p = 0.59
Right Hand Repeat: F (2, 54) = 0.63, p = 0.54
Right Hand Switch: F (2, 54) = 0.92, p = 0.40

Tests of normality:

RT-hysteresis: Shapiro-Wilk (57) = 0.99, p = 0.69

(S4-3) Right-handers No-strategy (N = 43): RM-ANOVA Hand (2) x History (2)

Main effect: Hand: F (1, 42) = 0.99, p = 0.33
Main effect: History: F (1, 42) = 17.0, p < 0.001
Interaction: Hand x History: F (1, 42) = 3.09, p = 0.09

Tests of normality:

RT-hysteresis: Shapiro-Wilk (43) = 0.97, p = 0.33

(S4-4) Right-handers No-strategy, outlier removed (N = 42): RM-ANOVA Hand (2) x History (2)

Main effect: Hand: F (1, 41) = 0.59, p = 0.45
Main effect: History: F (1, 41) = 41.0, p < 0.001
Interaction: Hand x History: F (1, 41) = 2.31, p = 0.14

Tests of normality:

RT-hysteresis: Shapiro-Wilk (42) = 0.98, p = 0.82

Table S5. Response Times: History by (PSE/Extreme) Target Position

(S5-1) Full dataset (N = 58): mixed ANOVA History (2) x Target Location (2) x Group (3)

Main effect: History: $F(1, 55) = 4.16$, $p < 0.05$
Main effect: Target Location: $F(1, 55) = 126.0$, $p < 0.001$
Main effect: Group: $F(2, 55) = 1.72$, $p = 0.19$
Interaction: History x Target Location: $F(1, 55) = 2.60$, $p = 0.11$
Interaction: History x Group: $F(2, 55) = 0.02$, $p = 0.98$
Interaction: Target Location x Group: $F(2, 55) = 0.02$, $p = 0.98$
Interaction: History x Target Location x Group: $F(2, 55) = 1.80$, $p = 0.18$

Box's test:

Box's M = 16.8, $F(20, 1018) = 0.64$, $p = 0.89$

Levene's test:

Extreme-Repeat: $F(2, 55) = 0.63$, $p = 0.54$
Extreme-Switch: $F(2, 55) = 1.17$, $p = 0.32$
PSE-Repeat: $F(2, 55) = 0.07$, $p = 0.94$
PSE-Switch: $F(2, 55) = 0.32$, $p = 0.73$

Tests of normality:

RT-hysteresis: Extreme: Shapiro-Wilk (58) = 0.99, $p = 0.85$
RT-hysteresis: PSE: Shapiro-Wilk (58) = 0.98, $p = 0.33$

(S5-2) Outlier removed: No outliers detected

(S5-3) Right-handers No-strategy (N = 43): RM-ANOVA History (2) x Target Location (2)

Main effect: History: $F(1, 42) = 4.96$, $p < 0.05$
Main effect: Target Location: $F(1, 42) = 154.2$, $p < 0.001$
Interaction: History x Target Location: $F(1, 42) = 0.53$, $p = 0.47$

Tests of normality:

RT-hysteresis: Extreme: Shapiro-Wilk (43) = 0.98, $p = 0.65$
RT-hysteresis: PSE: Shapiro-Wilk (43) = 0.97, $p = 0.42$

(S5-4) Right-handers No-strategy Outlier removed: No outliers detected

Table S6. Linear regression: Choice- and RT-hysteresis

(S6-1) Full dataset (N = 58)

ANOVA: $F (1, 56) = 6.33$, $p < 0.05$; $R^2 = 0.10$

Pearson Correlation = 0.32

Cook's distance, max = 0.22

Durbin-Watson = 1.80

Tests of normality:

RT-hysteresis: Shapiro-Wilk (58) = 0.98, p = 0.40

Choice-hysteresis: Shapiro-Wilk (58) = 0.93, p < 0.005

(S6-2) Outlier removed (N = 55)

ANOVA: $F (1, 53) = 6.19$, $p < 0.05$; $R^2 = 0.11$

Pearson Correlation = 0.33

Cook's distance, max = 0.22

Durbin-Watson = 1.73

Tests of normality:

RT-hysteresis: Shapiro-Wilk (55) = 0.99, p = 0.70

Choice-hysteresis: Shapiro-Wilk (55) = 0.97, p = 0.12

(S6-3) Right-handers No-strategy (N = 43)

ANOVA: $F (1, 41) = 5.10$, $p < 0.05$; $R^2 = 0.11$

Pearson Correlation = 0.32

Cook's distance, max = 0.27

Durbin-Watson = 1.88

Tests of normality:

RT-hysteresis: Shapiro-Wilk (43) = 0.97, p = 0.33

Choice-hysteresis: Shapiro-Wilk (43) = 0.92, p < 0.01

(S6-4) Right-handers No-strategy, outlier removed (N = 40)

ANOVA: $F (1, 38) = 4.42$, $p < 0.05$; $R^2 = 0.11$

Pearson Correlation = 0.32

Cook's distance, max = 0.44

Durbin-Watson = 1.77

Tests of normality:

RT-hysteresis: Shapiro-Wilk (40) = 0.98, p = 0.79

Choice-hysteresis: Shapiro-Wilk (40) = 0.97, p = 0.25

Table S7. Linear regression: PSE and Waterloo values

(S7-1) Full dataset (N = 58)

ANOVA: $F (1, 56) = 17.6$, $p < 0.001$; $R^2 = 0.24$

Pearson Correlation = -0.49

Cook's distance, max = 0.29

Durbin-Watson = 2.24

Tests of normality:

PSE: Shapiro-Wilk (58) = 0.92, $p < 0.005$

Waterloo: Shapiro-Wilk (58) = 0.96, $p < 0.05$

(S7-2) Outlier removed (N = 56)

ANOVA: $F (1, 54) = 20.48$, $p < 0.001$; R2 = 0.28

Pearson Correlation = -0.52

Cook's distance, max = 0.37

Durbin-Watson = 2.0

Tests of normality:

PSE: Shapiro-Wilk (56) = 0.94, $p < 0.01$

Waterloo: Shapiro-Wilk (56) = 0.95, $p < 0.05$

Partic No.: ____________ Age: ____ Sex: ____Initials: _____ Eye dom:_______
KickF:_______

Date of test:_____________ Phone Ear:______

Waterloo Handedness Questionnaire
Which hand would you use when you:

	Left always	Left usually	Equal	Right usually	Right always
1. Draw	-2	-1	0	1	2
2. Write	-2	-1	0	1	2
3. Hammer	-2	-1	0	1	2
4. Use a pencil rubber	--2	-1	0	1	2
5. Use a toothbrush	-2	-1	0	1	2
6. Hold a sewing needle	-2	-1	0	1	2
7. Cut bread with a knife	-2	-1	0	1	2
8. Swing a tennis racket	-2	-1	0	1	2
9. Throw a ball	-2	-1	0	1	2
10. Shave with a razor	-2	-1	0	1	2
11. Strike a match	-2	-1	0	1	2
12. Use a fly swatter	-2	-1	0	1	2
13. Use tweezers	-2	-1	0	1	2
14. Roll a marble	-2	-1	0	1	2
15. Use a comb	-2	-1	0	1	2

COLEG GWYDDORAU IECHYD AC YMDDYGIAD
COLLEGE OF HEALTH & BEHAVIOURAL SCIENCES

YSGOL SEICOLEG
SCHOOL OF PSYCHOLOGY

Experiment 001 Post-Experiment Questionnaire

(1) How did you decide which hand to use?

Please circle which of the following (a or b) best describes how you made your choice:

a) I used a specific strategy, or rule.

b) I didn't think much about it, I just responded.

(2) If you used a strategy, or rule, please describe what it was here:

(3) Please feel free to add any further comments, and/or suggestions:
